# Recurrent bladder leiomyoma: a case report

**DOI:** 10.1186/s13256-024-04372-y

**Published:** 2024-03-22

**Authors:** Fnu Yogeeta, Zubda Malik, Sameer Abdul Rauf, Muskan Devi, Fnu Tooba, Syed Abdan Jamalvi, Marium Rashid, Humaira Erum

**Affiliations:** 1 Department of Internal Medicine, Liaquat National Hospital and Medical College, Karachi, Pakistan; 2 Department of Urology, Liaquat National Hospital and Medical College, Karachi, Pakistan; 3 Department of Histopathology, Liaquat National Hospital and Medical College, Karachi, Pakistan

**Keywords:** Recurrent bladder tumor, Lower urinary tract symptoms, Painless hematuria, Transurethral resection

## Abstract

**Background:**

Bladder leiomyomas are rare benign growths in the bladder, comprising less than 0.5% of bladder tumors with only 250 cases reported globally. They are more common in women. This case involves a 70-year-old woman with recurrent leiomyoma, presenting with lower urinary tract symptoms and painless hematuria. A recurrent bladder leiomyoma is rarely reported, making its presence exceptionally rare.

**Case presentation:**

A 70-year-old Pakistani woman with hypertension and diabetes presented with lower urinary tract symptoms (LUTS) and painless hematuria. She had a history of similar symptoms in 2010, leading to a diagnosis of bladder leiomyoma via cystoscopy and biopsy. Imaging studies revealed a substantial 3.7 × 4 × 4.0 cm isodense mass with calcifications at the bladder base, along with bladder wall thickening and diverticula. Pathological examination during Transurethral Resection of Bladder Tumor (TURBT) confirmed the presence of bladder tissue with smooth muscle, ruling out malignancy. Immunohistochemical studies supported the diagnosis. A successful TURBT was performed, and the patient recovered well.

**Discussion:**

Recurrent bladder leiomyoma is a rarely-discussed topic in medical literature. This article primarily aims to review existing studies and present a detailed case study, shedding light on this rare phenomenon.

## Background

Bladder leiomyomas are uncommon benign growths of connective tissue in the bladder, accounting for less than 0.5% of all bladder tumors [1]. There have been only about 250 reported cases of this condition worldwide. Women are three times more likely than men to develop bladder leiomyomas due to factors like hormonal influence (higher estrogen levels) and anatomical differences in the urinary system [2, 3]. An awareness of this unusual tumor is important for urologists, who may encounter similar cases in their general urology practice.

We present a case of recurrent leiomyoma of the urinary bladder in a 70-year-old woman with typical symptoms of lower urinary tract symptoms and painless hematuria not associated with burning micturition, poor steam, nocturia, urgency or stress incontinence. She had a similar episode of symptoms in 2010, for which a cystoscopy plus biopsy was taken in 2012, which showed a leiomyoma spindle cell tumor.

## Case presentation

A 70-year-old Pakistani female with hypertension and diabetes mellitus presented to the urology outpatient department with complaints of Lower Urinary Tract Symptoms (LUTS) and painless hematuria for 1 week. The hematuria was gross and not associated with burning micturition, poor stream, nocturia, urgency, or urge/stress incontinence. She had a previous episode of similar symptoms in 2010 and underwent a cystoscopy in 2012, which revealed acute and chronic non-specific inflammation. The biopsy at that time showed bladder leiomyoma for which she had undergone Transurethral Resection of Bladder Tumor (TURBT) with no postoperative complication.

**Fig. 1:**
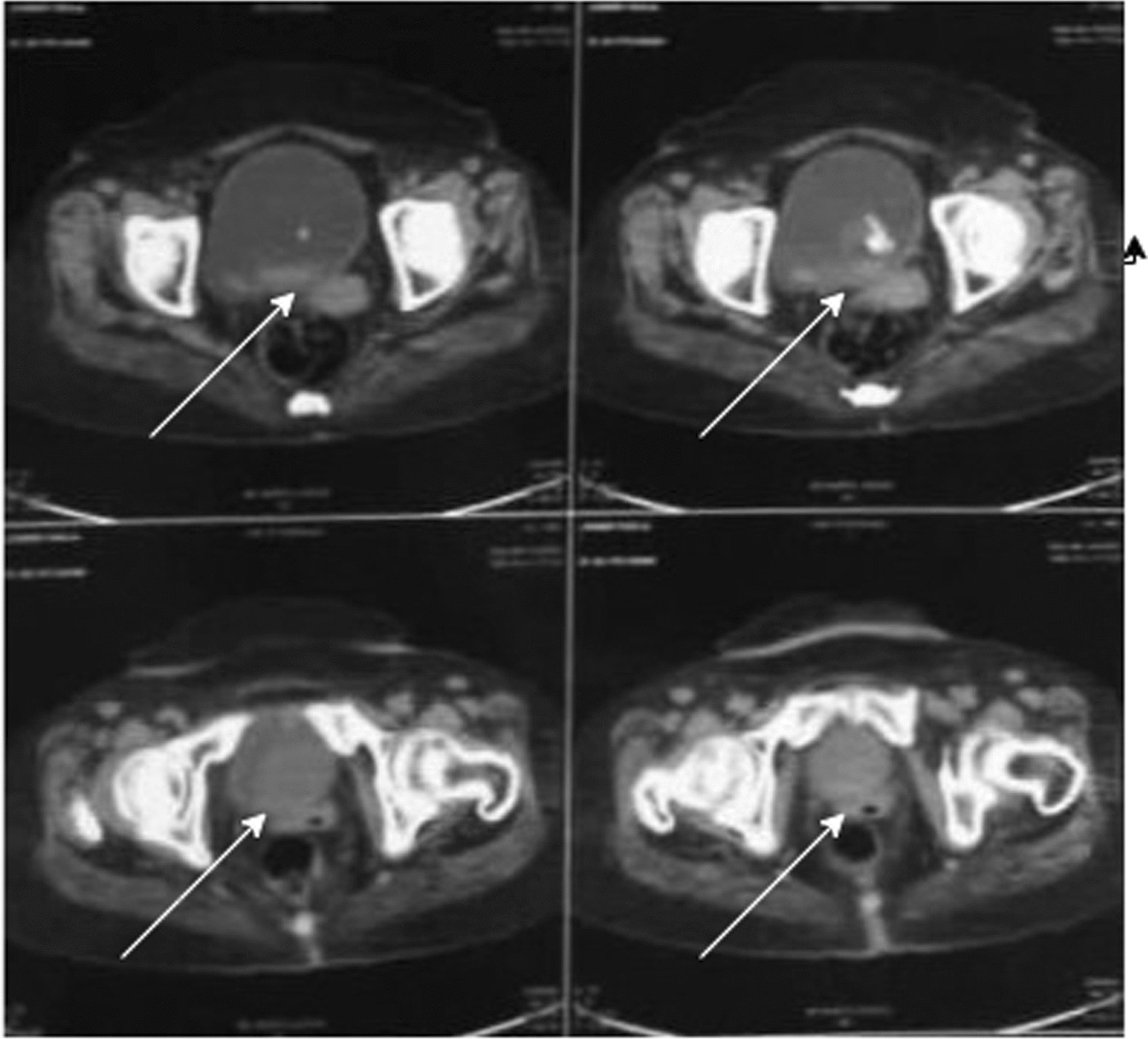
Arrows shows the presence of a sizeable mass, measuring approximately 3.7 × 4 × 4.0 cm, with calcifications around its outer edges and originating from the base of the urinary bladder

**Fig. 2:**
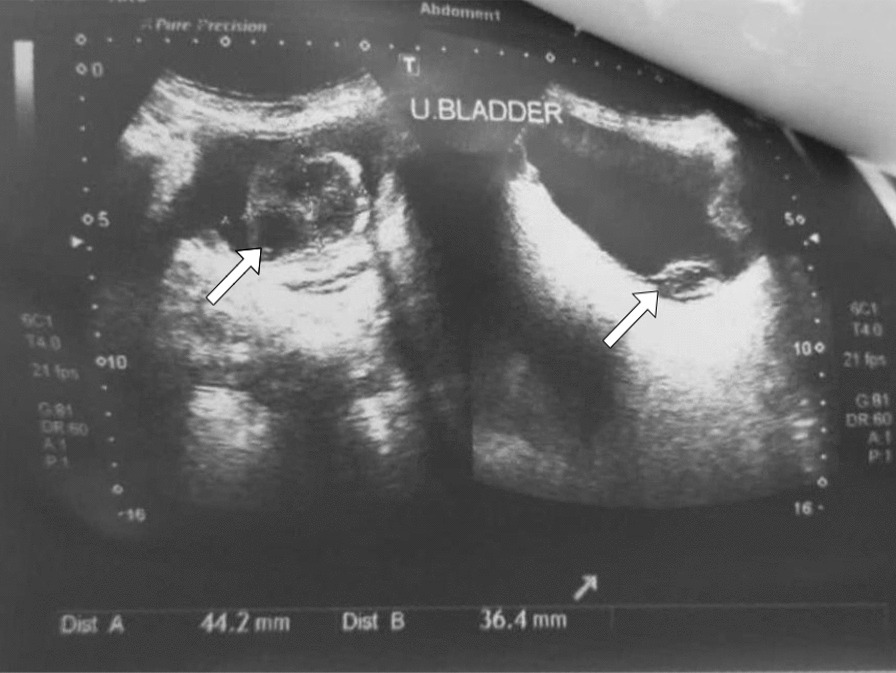
Arrows shows a 4.4 × 3.6 cm heterogeneous mass at its base, a 4.5 cm hypoechoic lesion within the bladder, and a diverticula

Upon initial assessment, her vital signs were stable. Physical examination revealed no signs of lymphadenopathy, suprapubic tenderness, or costovertebral angle tenderness, suggesting the absence of significant acute abnormalities or systemic involvement. Imaging studies, including a CT KUB (Computed Tomography of the Kidneys, Ureters and Bladder), revealed a substantial 3.7 × 4 × 4.0 cm isodense mass lesion with peripheral calcification originating from the base of the urinary bladder (Fig. 1). The scan also identified diffuse bladder wall thickening and a left lateral wall diverticulum. A whole abdomen ultrasound detected simple kidney cysts, a 4.4 × 3.6 cm heterogeneous mass at the urinary bladder’s base, another 4.5 cm hypoechoic lesion within the bladder, and diverticula (Fig. 2).

**Fig. 3:**
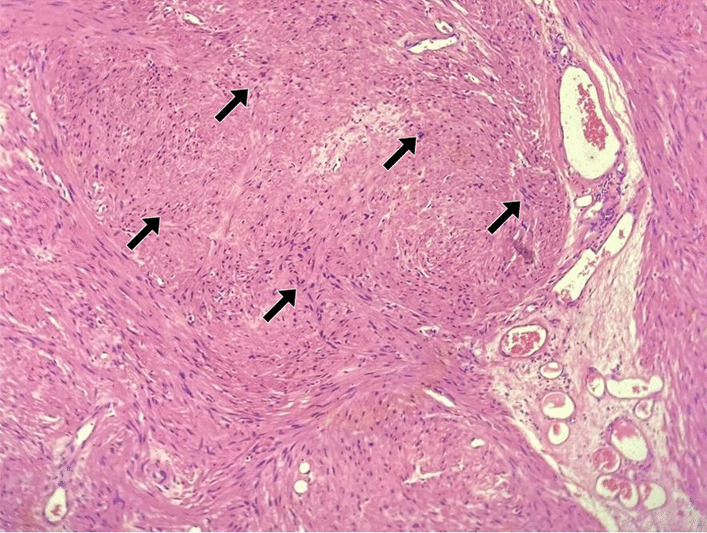
Arrows shows a neoplastic lesion beneath the urothelium composed of fascicles of spindle cells with bland nuclei

**Fig. 4:**
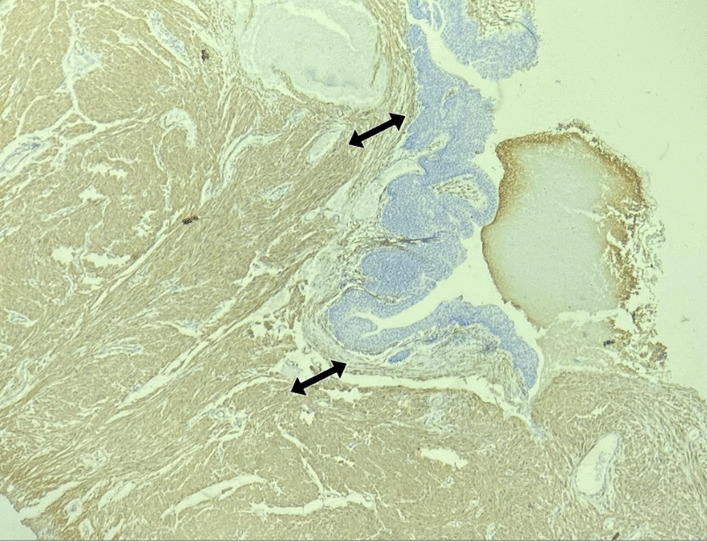
Arrows shows that Immunohistochemical stain smooth muscle actin (ASMA) is positive in spindle cells and negative in urothelium

Pathological examination during TURBT found bladder tissue with focal urothelial lining and smooth muscle, ruling out malignancy (Fig. 3). Immunohistochemical studies confirmed Anti-smooth muscle antibody (ASMA) antibodies positive and S-100 negative (Fig. 4). Transurethral Resection of Bladder Tumor (TURBT) was performed, and a large bladder tumor (5 × 6 cm) was resected. The patient tolerated the procedure well, and the catheter was removed on postoperative day 1. Table 1 highlights results of important laboratory investigations to reach a diagnosis.

**Table 1:** Re﻿sults of laboratory investigations

Laboratory investigations	Results	Normal reference range
1. Hematology		
Hemoglobin (gm/dl)	10.40	12–16
TLC	6.67	4.0–10
PCV (%)	43.00	36–54
Platelet count (× 10^9^/L)	160	140–440
PT (seconds)	12.50	11–13.5
INR	1.27	0.8–1.1
2. Renal profile		
Urea (mg%)	30.00	10–50
Creatinine (mg%)	0.73	0.5–1.5
Chloride (mmol/L)	103.00	0–111
Sodium (mmol/L)	137.00	137–150
Potassium (mmol/L)	3.90	3.5–5.3
Bicarbonate (mmol/L)	23.00	22–34
Uric Acid (mg/dl)	5.4	3.5–7.2
3. Urine DR		
PH	5.5	5–8
Glucose	+ 3	Negative
Blood	+ 3	Negative
Pus Cell	1–2	Negative
Epithelial	4	Negative
Urine C/S	Negative	Negative

TLC: Total Leukocyte Count, PCV: Packed Cell Volume, DR: Detail report

## Discussion

Bladder leiomyoma is a rare submucosal tumor with an incidence rate of less than 0.5% among all bladder growths [1]. The exact cause of these benign tumors is not yet fully understood. Some theories suggest that bladder leiomyomas could develop due to chromosomal changes, hormonal imbalances, recurrent bladder wall and detrusor infections, perivascular inflammation, or abnormal tissue development [4, 5]. They are believed to originate from smooth muscle fibers separated by connective tissue with low mitotic activity, necrosis, or cellular atypia [6]. They are classified into three types based on their location: endovesical, extravesical, and intramural [7, 8]. The endovesical type is the most common, while the intramural type is rare [9].

Clinically, leiomyomas of the bladder can be asymptomatic or present with complaints such as hesitancy, frequency, dribbling, hematuria, pressure from mass effect, and urinary obstruction [5, 9].

The diagnostic procedure for bladder leiomyoma involves imaging studies such as ultrasound, CT, and MRI to locate and characterize the tumor. Cystoscopy allows direct visualization, and if a suspicious lesion is found, a biopsy is performed for histological examination [10]. However, a biopsy study is considered the gold standard for diagnosis, as it confirms the presence of smooth muscle cells appearing as round nodules, grey-white, confirming the leiomyoma diagnosis [11]. Further imaging and evaluation may assess the extent of the tumor and exclude other conditions.

Management options for leiomyoma depend on the tumor size and anatomical location [12]. As a general guideline, transurethral resection of bladder tumors (TURBT) is typically suitable for managing small endovesical bladder leiomyomas [13, 14]. In contrast, larger intramural or extravesical tumors often require open surgical resection. It is crucial to ensure that any remaining tumor after surgery does not obstruct the ureteric orifice, as this could cause hydronephrosis [14, 15]. The recurrence rate of bladder leiomyoma is very rare and has never been reported [16].

## Conclusion

In conclusion, this case report discusses the rarity of Recurrent bladder leiomyomas, The study emphasizes the challenges in diagnosis and management, detailing the diagnostic process involving imaging, cystoscopy, and biopsy. Biopsy confirming smooth muscle cell presence is crucial for accurate diagnosis. Management varies based on tumor size and location, with TURBT suitable for small endovesical leiomyomas and open surgical resection for larger ones. The report notes the extremely rare recurrence rate, highlighting the importance of precise diagnosis and careful postoperative monitoring. The study underscores the need for continued research and awareness in urological practice.

## Data Availability

Not applicable.

## Abbreviations

LUTS: Lower urinary tract symptoms
TURBT: Transurethral resection of bladder tumor
CT KUB: Computed tomography of the kidneys, ureters and bladder
ASMA: Anti-smooth muscle antibody
CT: Computed tomography
MRI: Magnetic resonance imaging
